# Competent with patients and populations: integrating public health into a medical program

**DOI:** 10.1186/s12909-019-1635-6

**Published:** 2019-05-31

**Authors:** Colin Bell, Annie Simmons, Erik Martin, Candice McKenzie, Janet McLeod, Scott McCoombe

**Affiliations:** School of Medicine, Deakin University, Locked Bag 2000, Geelong, Victoria 3220 Australia

## Abstract

**Background:**

As the global burden of chronic disease grows, and infectious disease threats loom large, the need for medical graduates with expertise in public health medicine (PHM) is growing. A recurrent challenge is integrating this broad knowledge into crowded medical curricula and making PHM relevant. This study describes the process of integrating public health content into an Australian graduate entry medical course.

**Methods:**

A redesign of the PHM curriculum at Deakin University School of Medicine was conducted in 2014 to make the curriculum practice-based and solution-oriented. Central to the redesign was the development of a curriculum map.

**Results:**

Public health is now taught from a practice-based framework adapted from the World Health Organization emphasizing skills aligned with the Australasian Faculty of Public Health Medicine domains that prepare students for specialisation. Learning outcomes are structured to build depth and application in student knowledge. Mapping the curriculum provided the ability to measure alignment of learning outcomes with course, university and accrediting body outcomes. Regular feedback from students indicates engagement has improved along with perceived relevance to future careers.

**Conclusions:**

Doctors with public health skills are increasingly sought after in Australia, particularly in rural areas. Deakin graduates are well placed to meet this demand.

## Background

Public health medicine is the part of medical practice that tackles the health of populations. Over the last 30 years, the major trend in causes of death globally has transitioned from infectious diseases towards non-communicable diseases (NCDs) and injuries [1]. In Australia, ischaemic heart disease is the most common cause of death, followed by lung cancer, stroke and self-harm [2]. The determinants of these NCDs and injuries are complex and in many cases, sit beyond the reach of health services. While infectious diseases are no longer the major cause of death in Australia, the threat of illness and death on a large scale is ever present as bacteria and viruses evolve [3], and as the likelihood of exposure to novel pathogens increases with international travel [4].

For these reasons, and after a long period where the relevance of public health in medicine was questioned [5–7], contemporary medical education is embracing the principles of public health and preventive medicine [8]. Once again, Rose’s call to prioritise prevention [9], has been heeded and work-ready physicians who can realize opportunities to prevent disease and control burgeoning health system costs throughout their career are in demand [10]. Respected medical programs recognize this and include public health in their course content [11], or through offering joint Master of Public Health/Medicine courses [12]. Further, doctors in Australia need to be at the forefront of addressing health inequities impacting rural, remote, Indigenous and marginalised individuals and communities [13, 14].

The Australian Medical Council (AMC), accredits medical education providers in Australia and New Zealand [15]. The council recognizes the need for graduates to have public health skills under the nine outcomes of the ‘health and society’ domain. Importantly, skills and knowledge in public health medicine are also recognized under the other three AMC domains that envisage graduates as ‘scientists and scholars’, ‘Clinical practitioners’ and ‘Professionals and Leaders’.

The School of Medicine at Deakin University is the state of Victoria’s first rural and regional medical school. The school accepted its first students in 2008 and has a vision of equipping students with the education and experience necessary to become professional and work-ready medical practitioners. PHM contributes 12.5% of this four-year, graduate entry Doctor of Medicine (MD) course.

One of the challenges facing PHM staff is making this diverse component of the course medically relevant and interesting for students. Perhaps influenced by media portrayals [16], and by their own experiences of doctors (applicants often have parents or family members who are medical doctors [17]) many students arrive at medical school with predetermined and sometimes narrow views of what it takes to be a doctor. PHM does not usually feature in the prevailing student view and for many students is a hard ‘sell’ because it is considered ‘soft’ and too broad.

We describe here a redesign and enhancement of a compulsory part of the medical curriculum. The purpose of this research is to articulate a clear curriculum framework for the PHM theme that could be adopted and adapted by other medical programs. Further, to provide transparency on how well the course aligns with global calls to incorporate PHM, current AMC standards and outcomes and a benchmark against which quality improvements can be made in the future.

## Methods

We set out to develop an evidence informed curriculum framework that was practice-based and solution-oriented. Secondly, to produce a curriculum map or blueprint that enhanced constructive alignment between learning outcomes, activities and assessment as well as alignment with mandatory AMC standards and outcomes and Australasian Faculty of Public Health Medicine (AFPHM) advanced training curriculum themes. AFPHM themes are not mandatory but basic training in these themes keeps the curriculum practice-based and prepares students for future specialization in PHM in Australia or New Zealand. Finally, to identify clinically relevant content and delivery mechanisms that build relevance and interest for students and improve student feedback.

### Evidence-informed curriculum framework

In 2013, a curriculum redevelopment process was initiated to identify a new curriculum framework for teaching PHM at Deakin. The process involved four phases: 1) a narrative review of published and grey literature on public health teaching and learning frameworks and contemporary medical teaching; 2) a series of workshops on framework concepts and ideas involving course staff, medical students and public health practitioners; 3) in 2014; discussion and refinement of a framework with student representatives during the first year of delivery; and 4) course executive approval of the framework.

### Detailed curriculum mapping

The PHM curriculum framework enabled mapping of the teaching and learning outcomes, graduate outcomes and professional attributes across all 4 years of the course. This mapping exercise was conducted during 2014 and the first full spreadsheet version of the curriculum was available at the end of that year. Coordinators of each of the year levels of PHM entered teaching and learning activities into the spreadsheet and aligned these with learning outcomes and AMC attributes. For each student interaction, information was recorded on the type of learning activity provided (lecture, team-based learning activity, assessment, field trip, tutorial), learning outcomes were developed and refined, hours of staff input captured, and required associated learning activities recorded (readings, self-reflection, online resources). The map captures alignment with AMC domains [15], AFPHM advanced training curriculum themes [18], unit learning outcomes, course learning outcomes, Deakin University graduate learning outcomes and key words associated with the activity. The total number of learning outcomes in the PHM curriculum was then added up and the proportion that meet AMC domains, AFPHM themes and Deakin graduate learning outcomes was calculated.

### Improving student feedback

We used formal and informal evaluation data from students to determine if the introduction of the new curriculum framework and mapping process improved learning outcomes and made the course more relevant and interesting. As teaching staff, the authors had access to student feedback on PHM teaching in the medical course. This data was collected anonymously from students at the end of each semester from 2008 to 2016. We captured the percentage of students who agreed or strongly agreed with the statement that the PHM unit was taught well (2008–2014) or that ‘the quality of teaching in this unit helps me to achieve the learning outcomes’ (2015, 2016). Data are provided for student’s experience of semester one in year one of their course. Also, each year, students provide an independent report to the Australian Medical Council. The 2014 and 2015 reports were reviewed for comments relevant to PHM.

## Results

### Redesigning the curriculum: framework

**Fig. 1:**
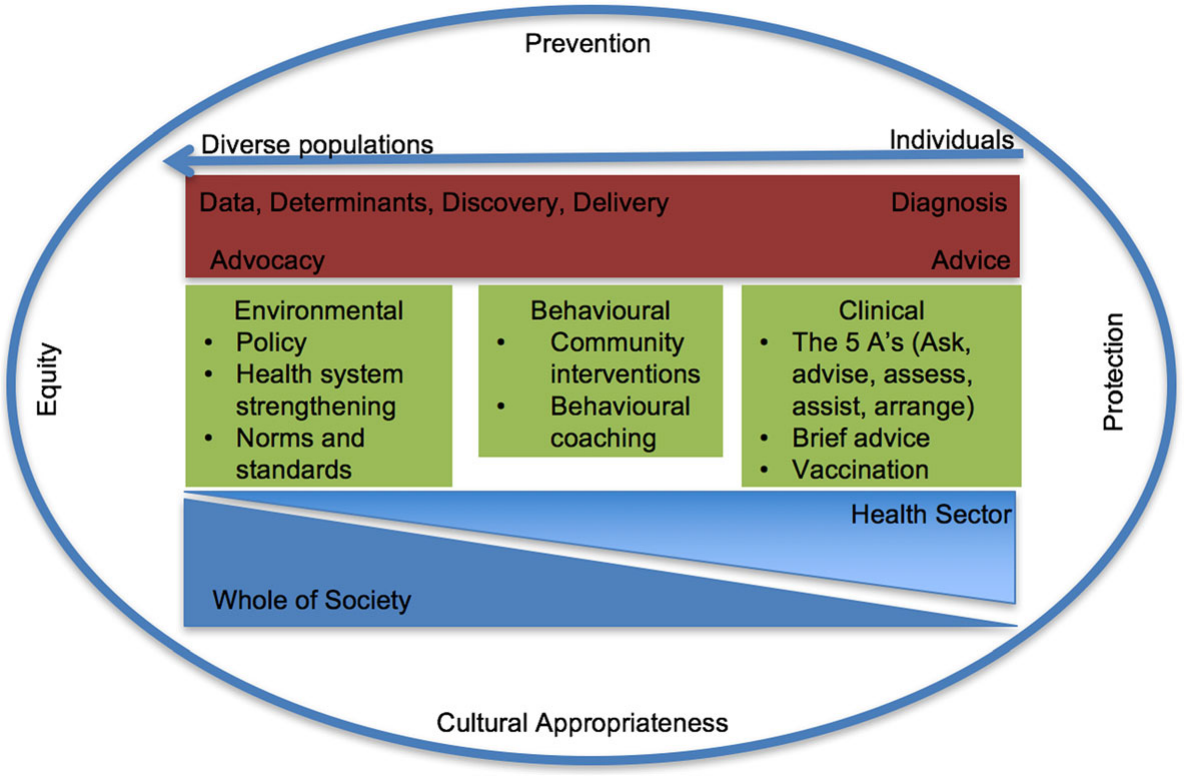
Curriculum framework

Our review revealed that existing teaching frameworks tend to be centered on the theory, science and disciplines of broad public health [19, 20], or competencies [21], rather than medical practice. For example, as a key discipline of PHM, epidemiology is often taught in a block and introduced from the perspective of how the discipline emerged [22], rather than how medical practitioners use it as a tool to help patients or populations. In our view, medical graduates need to know what the burden of disease will look like when they graduate, what the major threats to health are, and what the most effective ways are to respond to them given the confines of medicine and health systems. Thus, we adapted a practice-based framework for action on non-communicable diseases [23], to form the curriculum framework (Fig. 1).

Pervading all teaching content are principles of prevention, equity, cultural appropriateness and protection. These principles are taught in a spiral pattern so they are covered multiple times, in multiple ways, and with increasing depth and application and are experiential where possible. For example, cultural appropriateness is introduced through a 3-day cultural immersion program in year 1 and skills are developed in subsequent years through interaction with Aboriginal Health staff, discussions with experts on Indigenous Knowledges and placement in Aboriginal Health Services. The framework recognises the diagnosis and treatment of conditions in individuals as central to medical education (upper right of the framework) but the framework includes populations (local and global) in the scope of care doctors provide. This ensures we train aspiring doctors to look beyond the individual patient and develop their competence in population approaches to prevention [9].

### PHM skills

Consistent with the practical nature of the framework, the first section (red in Fig. 1) identifies the skills we believe doctors need to provide care for individuals and populations. Relevant skills include the ability to critically appraise *data* (epidemiology and biostatistics); identify and map causal pathways and the *determinants* of health; *discover* answers to public health medical questions through research; *deliver* quality health care solutions (preventive medicine and public health). Aligned with the more clinical components of the course, students also learn how public health tools underpin accurate *diagnosis* in individuals, so they can manage, treat and prevent health conditions more effectively.

The communication skills emphasized in the framework are the ability to provide brief *advice* to the individual and to *advocate* for the rights of patients and health equity in communities. Acquiring these skills begins in the first week of the course where students learn the major causes of death and disease within and beyond Australia and how determinants impact individuals and populations. As disease patterns emerge, we introduce the students to epidemiological units of measure such as mortality rates, incidence, prevalence and Disability-Adjusted-Life-Years (DALYs). In this way, the framework taps into a strong motivation for studying medicine (improving health) while providing a platform for students to learn the fundamentals of PHM and why they are useful.

### PHM solutions

The framework also helps us teach students the unique solutions and interventions that PHM can offer for identifying high risk individuals and delivering care. These are grouped into *‘Clinical’*, *‘Behavioural’* and *‘Environmental’* interventions (green in Fig. 1). These interventions target the causes of morbidity and mortality in the Australian context. They draw on relevant research, patient journey’s and clinical examples so that students can appreciate the influence PHM interventions have had in the past (for example reducing cardiovascular disease mortality or controlling communicable diseases using vaccines) and how they can be applied to current epidemics such as obesity. Students screen each other for NCD risk factors, calculate their absolute CVD risk and analyse and discuss class-level risk in lectures. As the interventions span individuals through to populations, they naturally draw students upstream to the social determinants of health, or unseen parts of the iceberg of health first proposed by Logan [24].

Finally, by demonstrating that prevention and control of health conditions requires clinical, behavioural and environmental intervention, students become adept at big-picture thinking and develop an appreciation for the complexity of disease causation and the need for multi-strategy solutions.

### PHM partners

Lastly, the framework helps students recognise that public health practitioners cannot work in isolation and that partners, within and beyond the health sector, are crucial for a highly functioning health system (blue in Fig. 1). The wedges indicate that as health care moves further away from the individual and towards the larger population, the contribution of the *‘health sector’* diminishes and the contribution of other sectors in the *‘whole of society’* increases. For example, a doctor providing brief advice to a patient to quit smoking fits in the role and function of the *‘health sector’*. Alternatively, instigating a policy for smoke-free environments involves a broad range of experts, advocates and community members drawn from the *‘whole of society’*. Students are exposed to practitioners in the health sector including surgeons, general practitioners, health service chief executive officers, Doctors working in Aboriginal Controlled Community Health Organisations, Chief Medical Officers and Global Health Experts. They are also exposed to practitioners outside the formal health sector ranging from those running community kitchens to local NGOs providing housing for refugees to global NGO’s helping low and middle-income countries achieve development goals.

### Curriculum

The PHM curriculum lists the learning outcomes taught in PHM arranged by teaching and learning activity (Table 1).

**Table 1:** Excerpt from preclinical (year 2) section of the Public Health Medicine curriculum blueprint

Learning Activity	Lecturer	Title	Learning Outcomes	Learning Materials	AMC Domain	RACP PHM ATC Theme	Deakin GLO	Assessment	Key words
Year 2									
Lecture	EM	Introduction to PHM & Public Health research	1. Describe the scope, rationale, fundamental components, and solution-oriented approach of Public Health Medicine at Deakin University 2. Describe the context and underpinnings of public health research and how this differentiates from traditional epidemiological research	Baum (2008) Chapter 6 ‘Research for a New Public Health’ of *The New Public Health*	1.1, 1.4	3.2	1, 8	Expression of Interest	Research, epistemology, old/new public health
Lecture	EM	Qualitative research & Research translation	1. Distinguish qualitative and quantitative methodologies and recognise their appropriate use 2. Identify and explain common qualitative research methods 3. Explain how research findings can be translated into practice in clinical and public health settings 4. Demonstrate an understanding of scientific and medical research process and explain translational research	Baum (2008) Chapter 9 ‘Qualitative Research Methods’ of The New Public Health	1.2, 1.4, 1.5	3.1, 3.2	1, 4, 5	Expression of Interest	Qualitative research methods, research translation, translational research

*RACP PHM ATC* Royal Australasian College of Physicians Public Health Medicine Advanced Training Curriculum, *AMC* Australian Medical Council, *GLO* Graduate Learning Outcome. Expression of Interest – a 2000 word written assessment on a community health topic

Across the course, the PHM team delivers 401 distinct learning outcomes via 114 face-to-face and online learning activities. With a map of the range of learning outcomes and teaching and learning modalities we can estimate depth of and/or gaps in student learning. For example, teaching and learning outcomes on public health advocacy step up through the Structure of Observed Learning Outcomes (SOLO) taxonomy [25], traversing multiple year levels and delivered through varying modalities including lecture, media interviews, online learning modules using online learning management system, free online resources, library resources and Youtube videos. Furthermore, we can capture integration into other parts of the course. For example, some PHM learning outcomes are integrated into patient scenarios delivered through Problem-Based Learning.

**Fig. 2:**
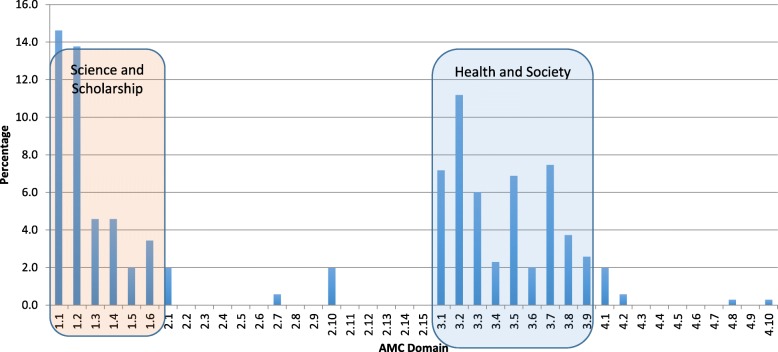
Proportion of Public Health Medicine outcomes aligned with Australian Medical Council (AMC) domains

The PHM curriculum also indicates how and where in the course particular learning objectives are assessed. Advocacy, for example, is assessed via multiple choice questions, short answer questions, online quizzes, and at the extended abstract level [25], via a written advocacy piece. Finally, the PHM curriculum map also indicates if a learning objective is assessed. For example, where learning outcomes are ‘orphaned’ and do not have clear alignment with an assessment task, this can be addressed in the following year of the course. Figure 2 shows the alignment of PHM curriculum with the AMC standards and outcomes.

The AMC domain under which most PHM teaching and learning occurs is domain 3 - ‘Health and Society’. We have learning outcomes covering all the standards in this domain and just under half (49.3%) of our learning outcomes were categorized within this domain. The figure also shows that most of our remaining learning outcomes align with the ‘Science and Scholarship’ domain (45%) and in particular, that students demonstrate an understanding of established and evolving biological, clinical, epidemiological, social, and behavioural sciences (14.1%) and apply core medical and scientific knowledge to individual patients, populations and health systems (13.8%). Domain 2 ‘Clinical Practice’ and Domain 4 ‘Professionalism and Leadership’ are covered by other themes in the course.

**Fig. 3:**
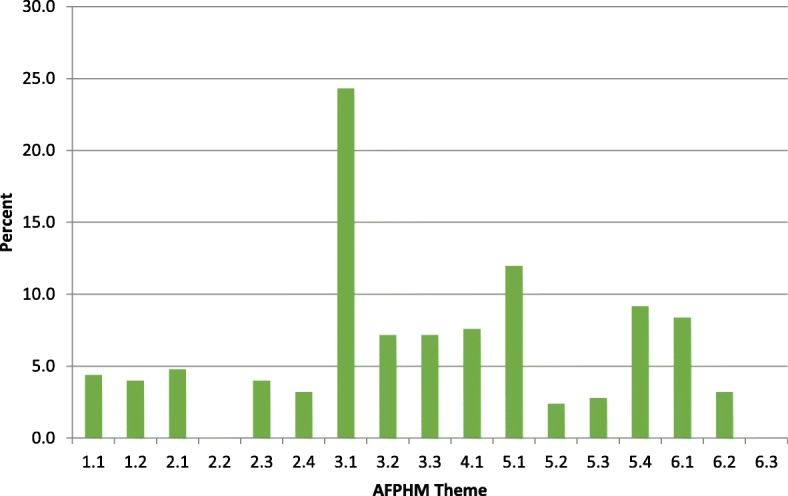
Proportion of public health medicine learning outcomes aligned with Australasian Faculty of Public Health Medicine (AFPHM) Advanced Training Curriculum themes

As AMC standards are relatively non-specific, to ensure coverage of the fundamentals of contemporary PHM and to provide basic training for future specialization, the PHM curriculum map learning outcomes were also aligned with the AFPHM Advanced Training Curriculum (Fig. 3).

**Fig. 4:**
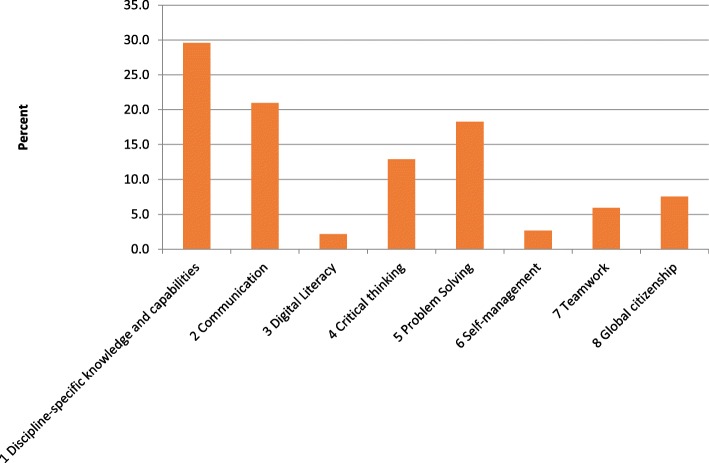
Proportion of Public Health Medicine learning outcomes aligned with Deakin’s Graduate Learning Outcomes

The figure indicates that over 20% of our teaching and learning outcomes in PHM relate to public health information and critical appraisal (Theme 3.1). This high percentage highlights the emphasis in the curriculum on the fundamentals of public health medicine. However, between 2 and 12% of our learning outcomes also cover the other themes including basic training so students can provide advice in rural and remote areas (Theme 6.2). The curriculum also helps us align our learning outcomes with the graduate learning outcomes of the University (Fig. 4) with PHM covering all eight graduate attributes at some level.

### Student feedback

**Fig. 5:**
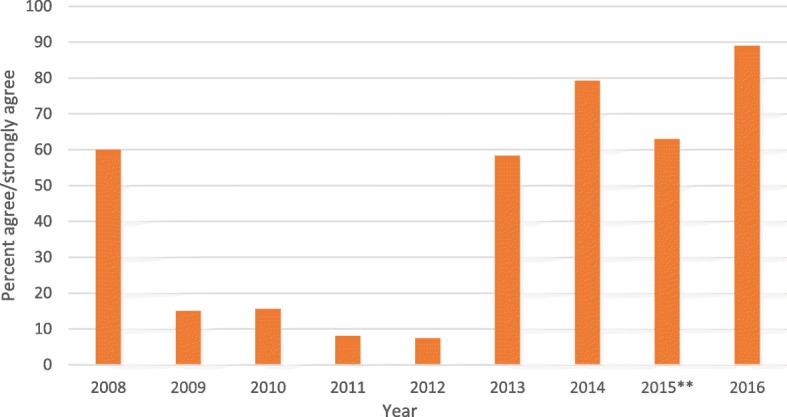
Student agreement that Public Health Medicine was taught well (2008 to 2014) or that the quality of teaching enhanced learning (2015–2016). ** Questions evaluating student feedback changed in 2015

Prior to the introduction of the new curriculum framework and PHM curriculum map, student feedback across all 4 years for the PHM theme was poor. Student feedback scores fell well below those of other themes within the medical degree, as well as below Faculty of Health and University-wide averages. Students reported that PHM content was lecture heavy as well as too theoretical and not relevant to future medical practice. Figure 5 shows that student feedback regarding quality of teaching in year one and semester one of the course has improved substantially since 2012 although it should be noted that response rates were low (~ 33%).

Since 2014 with the establishment of the new PHM curriculum, strategic recruitment of staff and visiting lecturers and a second iteration of refinement in 2015, PHM content now performs near the top of student feedback metrics within the medical degree. Indeed, the 2014 student submission to the Australian Medical Council stated:

*‘the curriculum framework of PHM has undergone significant review in recent years. The response of the school to student feedback has been greatly appreciated and positive steps have been made to increase the relevance of the subject to the role of a medical professional’* [26].

These sentiments were reiterated in the 2015 submission where it was also noted that ‘there are currently no major issues reported by students for this theme (PHM)’, and that it ‘provided students with opportunities to choose from a selection of topics in the pre-clinical years’ and that the ‘School of Medicine’s increasingly active engagement with student feedback, most notably in the PHM theme, is commendable’ [27].

## Discussion

The aim of the PHM curriculum redesign and mapping was to improve student learning and relevance while strengthening alignment with AMC, AFPHM, and University learning outcomes. Changes targeted improvements in the quality and modality of teaching, appropriateness of assessment and applicability for tomorrow’s doctors. Acknowledging that there is always room for improvement, the results presented in this paper indicate that the curriculum now covers most domains expected by the AMC and many of the AFPHM Advanced Training Curriculum themes. For example, we know that students are learning more about conditions that are National Public Health Priorities than they were and we can continue to monitor alignment [28]. Furthermore, feedback from students indicates they find the material interesting and relevant to their expectations of the role of a medical professional.

Medical education literature continues to debate whether or not to integrate public health into medical curricula and how this can best be achieved [29–31]. In his 1994 article, Woodward presents a case for and against the integration of public health into medical training. [32] Against integration, he argues that the ‘clinical imperative (i.e. diagnosis and treatment) is so firmly entrenched in the minds of students and in the cultures of medical schools that public health will always be diminished and elbowed to one side in medical curricula’ [32]. For integration, he argues that ‘the health of populations will not be improved without participation of all groups with an interest in, and an influence on, health care and that the medical profession is a particularly influential group’ [32]. This paper indicates that integration is possible. Gillam and Maudsley state that sharing educational goals, frameworks and good practice should promote better public health education, as well as better social accountability while heeding local circumstances [33]. It is for this reason that we describe our PHM framework and map for consideration by others in the field. Ultimately, this line of enquiry is based on the premise that better public health education for medical students makes better doctors [15, 34, 35].

The curriculum map has allowed us to look at the range of teaching and learning activities we offer and determine success in presenting concepts and content in public health medicine in ways that are consistent with student learning styles [36], and that provide ‘transforming knowledge, helping students to interpret and to construct their own knowledge’ [37]. Initial student feedback suggests that we have moved in the right direction [38]. It has also provided us with a tool to measure alignment of intended learning outcomes with teaching and learning activities and assessment tasks [39]. Boud sums up the value of constructive alignment well by contending that ‘learning should be worthwhile and that assessment should enhance worthwhile learning’. [40] We now know what we teach and how we are enhancing it with assessment tasks.

A strength of this paper is that it brings transparency and accountability to our teaching. It also gives us a baseline against which we, and others, can benchmark progress. A limitation is that the results provide only a short-term perspective on how changes have been received by students. Also, the university wide feedback tool we used to capture student feedback was relatively blunt, particularly pre-2015, meaning a more nuanced evaluation was not possible. Finally, Figs. 2, 3, 4 provide a perspective on the breadth of the curriculum but not the depth. For example, the figures do not capture the depth of teaching on Aboriginal and Torres Strait Islander health (3.4 in Fig. 2 and 3.2 in Fig. 3). Further research using data from university surveys and semi-structured interviews would allow further insight on both deep learning and student engagement. As the PHM curriculum map becomes more sophisticated, research will help us identify what threshold concepts exist in public health medicine and how best to teach them. Threshold concepts are only just starting to be explored in medical education and early studies have pointed to population perspectives as a likely threshold concept for learners [41]. Another may well be Indigenous Health and cultural competence.

## Conclusion

A strategic and evidence-based Public Health Medicine curriculum framework and detailed Public Health Medicine curriculum map have led to improved learning outcomes and alignment with professional body standards. The refined content and delivery have been favourably received by students. The full impact of these changes remains to be determined however, early findings are promising and should help Deakin University graduates meet Australia’s growing need for public health medicine savvy doctors [42].

## Data Availability

The dataset supporting the conclusions of this article is available from the corresponding author on request.

## Abbreviations

AFPHM: Australasian Faculty of Public Health Medicine
AMC: Australian Medical Council
DALYs: Disability Adjusted Life Years
MD: Doctor of Medicine
NCDs: Non-communicable diseases
PHM: Public Health Medicine
SOLO: Structure of Observed Learning Outcomes

## References

[1] GBD 2013 Mortality and Causes of Death Collaborators (2015). Global, regional, and national age–sex specific all-cause and cause-specific mortality for 240 causes of death, 1990–2013: a systematic analysis for the Global Burden of Disease Study 2013. Lancet.

[2] Alexandra N, Nowbar JP, Howard JA, Finegold PA, Francis PD (2014). Global geographic analysis of mortality from ischaemic heart disease by country, age and income: Statistics from World Health Organisation and United Nations. Int J Cardiol.

[3] Mahan MJ, Kubicek-Sutherland JZ, Heithoff DM (2013). Rise of the microbes. Virulence..

[4] Harvey K, Esposito DH, Han P, Kozarsky P, Freedman DO, Plier DA, Sotir MJ (2013). Surveillance for travel-related disease-GeoSentinel surveillance system, United States, 1997-2011. MMWR Surveill Summ.

[5] Kelishadi R. To the readers. Int J Prev Med. 2010;1(1):i. PMC3075481 21677759

[6] Loh LC, Peik SM (2012). Public health physician specialty training in Canada and the United States. Acad Med.

[7] Dickson EC (2000). Teaching of public health and preventative medicine. Acad Med.

[8] Ruis AR, Golden RN (2008). The schism between medical and public health education: a historical perspective. Acad Med.

[9] Rose G (2001). Sick individuals and sick populations. Int J Epidemiol.

[10] Monroe JA (2011). Exploring the context: contemporary public health. Am J Prev Med.

[11] University of Cambridge. Cambridge, UK: Medicine at Cambridge. http://www.undergraduate.study.cam.ac.uk/courses/medicine. Accessed 7 July 2017

[12] University of North Carolina. Chapel Hill, NC: MD-MPH program. http://www.med.unc.edu/www/education/combined-programs/md-mph 2016. Accessed 7 July 2017.

[13] Commonwealth of Australia. Department of the Prime Minister and Cabinet. Closing The gap. Prime ministers report. Canberra: Commonwealth of Australia; 2017.

[14] Godding Robyn (2014). The persistent challenge of inequality in Australia's health. The Medical Journal of Australia.

[15] Australian Medical Council Limited. Accreditation Standards for Primary Medical Education Providers and their Program of Study and Graduate Outcome Statements. Melbourne: Australian Medical Council; 2012.

[16] Tapper EB (2010). Doctors on display: the evolution of television's doctors. Proc Bayl Univ Med Cent.

[17] Simmenroth-Nayda A, Görlich Y (2015). Medical school admission test: advantages for students whose parents are medical doctors?. BMC Med Educ.

[18] The Royal Australasian College of Physicians. Public health medicine advanced training curriculum: Australasian Faculty of Public Health Medicine. Melbourne: The Royal Australasian College of Physicians; 2013.

[19] World Health Organization (2009). Teaching of public health in medical schools. Report of the regional meeting.

[20] Gillam S, Maudsley G. Public health education for medical students: a guide for medical schools. Newcastle-upon-Tyne: Cambridge University Press; 2008.

[21] Kaprielian VS, Silberberg M, McDonald M (2013). Teaching population health: a competency map approach to education. Acad Med.

[22] Rothman KJ, Greenland S (1998). Modern epidemiology.

[23] World Health Organization (2014). Western Pacific Regional Action Plan for the prevention and control of non-communicable diseases.

[24] Lachlan GM, Problems and Progress in Medical Care (1966). The Milbank Memorial Fund quarterly Vol. 44, no. 3, Part 1.

[25] Biggs J, Collis KF (1982). Evaluating the quality of learning - the SOLO taxonomy.

[26] Deakin Medical Students Association. Student Submission Australian Medical Council. 2014 Progress report. Geelong: Deakin University; 2014.

[27] Deakin Medical Students Association. Student Submission Australian Medical Council. 2015 Progress report. Geelong: Deakin University; 2015.

[28] Australian Institute of Health and Welfare. National Public Health Priority Areas. http://www.aihw.gov.au/national-health-priority-areas/. Accessed 29 Mar 2019.

[29] Jefferys M, Lashof J, Fee E, Acheson RM (1991). Preparation for public health practice: into the twenty-first century. A history of education in public health: health that mocks the doctors’ rules.

[30] Meshiro R (2010). Medical education for a healthier population: reflections on the Flexner report from a public health perspective. Acad Med.

[31] Mahoney JF, Fox MD, Chheda SG (2011). Overcoming challenges to integrating public and population health into medical curricula. Am J Prev Med.

[32] Woodward A (1994). Public health has no place in undergraduate medical education. J Public Health Med.

[33] Gillam S, Maudsley M (2009). Public health education for medical students: rising to the professional challenge. J Public Health.

[34] Medical Schools Council (MSC). The consensus statement. The Role of the Doctor: Past, Present and Future. London: MSC. Available from: http://www.medschools.ac.uk/ABOUTUS/PROJECTS/Pages/The-Role-of-the-Doctor.aspx. [2008, cited 2017 March 1]

[35] Black E (2016). Why isn’t learning about public health a larger part of becoming a doctor? The conversation.

[36] McInnes D (2013). The performance of academic identity as pedagogical model and guide in/through lecture discourse. Teach High Educ.

[37] Biggs J (1999). Teaching for quality learning at university.

[38] Sharma Rajendra (2008). The Australian perspective: Access, equity, quality, and accountability in higher education. New Directions for Institutional Research.

[39] Biggs J, Tang C. Applying constructive alignment to outcomes-based teaching and learning. Hobart; 2009. https://teaching.yale-nus.edu.sg/wp-content/uploads/sites/25/2017/03/biggs.tang_.constructive.alignment.What-is-CA-biggs-tang.pdf.

[40] Boud D. Assessment and learning – unlearning bad habits of assessment. Presented to the conference ‘effective assessment at university’. Queensland: University of Queensland; 1998. p. 4–5.

[41] Neve Hilary, Wearn Andy, Collett Tracey (2015). What are threshold concepts and how can they inform medical education?. Medical Teacher.

[42] Medical Deans Australia and New Zealand Media Release. Australia needs more doctors in the bush; not another urban medical school: Medical Deans Australian and New Zealand; 2015. http://www.medicaldeans.org.au/australia-needs-more-doctors-in-the-bush-not-another-urban-medical-school/. Accessed 7 July 2017

